# Prediction of Delirium in ICU Patients and Development of a CDSS Model Using Clinical Data Warehouse

**DOI:** 10.1093/geroni/igaf122.3997

**Published:** 2025-12-31

**Authors:** Sun Ju Kim

**Affiliations:** Chungnam National University Se-Jong Hospital, Se-Jong, Ch’ungch’ong-namdo, Republic of Korea

## Abstract

Delirium in intensive care unit (ICU) patients is a critical condition associated with prolonged hospital stay, increased mortality, and higher healthcare costs, underscoring the need for early prediction and prevention. This study aimed to identify risk factors for ICU delirium using a Clinical Data Warehouse (CDW) and to develop a Clinical Decision Support System (CDSS) integrating machine learning models. A retrospective cohort study was conducted on adult patients (≥18 years) admitted to the ICU of a tertiary hospital in Daejeon, South Korea, from May 2015 to May 2024. Patients were classified into two groups: those who developed delirium within five days of ICU admission and those who did not. Predictive models were constructed using logistic regression, decision tree, and XGBoost. Variables included demographics, biometric indicators, vital signs such as Glasgow Coma Scale (GCS), blood pressure, oxygen saturation, and past medical history. The decision tree model demonstrated high sensitivity, while the XGBoost model achieved the highest accuracy (0.66) and specificity. Logistic regression offered interpretability but showed limited sensitivity (0.22). Key predictive variables were GCS-Verbal, GCS-Eye, age, blood pressure, and oxygen saturation. Based on these findings, a CDSS was designed to proactively assess the risk of delirium in ICU patients. The results suggest that machine learning-based models, particularly XGBoost, have potential clinical utility for early detection and intervention strategies. Future research should focus on optimizing predictive models and evaluating real-time implementation to support clinical decision-making and improve outcomes for critically ill patients.

